# Anaesthetic Adverse Events During Propofol-Based Sedation for ERCP: A Real-World Cohort Study

**DOI:** 10.3390/jcm15103679

**Published:** 2026-05-11

**Authors:** Sonia Elena Popovici, Stelian Adrian Ritiu, Bogdan Miutescu, Tudor Voicu Moga, Iulia Ratiu, Ioan Sporea, Dorel Sandesc, Ovidiu Bedreag, Marius Păpurică, Raluca Lupusoru, Alina Popescu

**Affiliations:** 1 Faculty of Medicine, Victor Babes University of Medicine and Pharmacy Timisoara, 300041 Timisoara, Romania; popovici.sonia@umft.ro (S.E.P.); bedreag.ovidiu@umft.ro (O.B.); popescu.alina@umft.ro (A.P.); 2 Doctoral School, Victor Babes University of Medicine and Pharmacy Timisoara, 300041 Timisoara, Romania; 3 Anaesthesia and Intensive Care Research Center (CCATITM), Victor Babes University of Medicine and Pharmacy Timisoara, 300041 Timisoara, Romania; 4 Advanced Regional Research Center in Gastroenterology and Hepatology, Department VII: Internal Medicine II, Discipline of Gastroenterology and Hepatology, Victor Babes University of Medicine and Pharmacy Timisoara, 300041 Timisoara, Romania; isporea@umft.ro (I.S.); raluca.lupusoru@umft.ro (R.L.); 5 Clinic of Anaesthesia and Intensive Care, Emergency County Hospital “Pius Brînzeu”, 300723 Timisoara, Romania; 6 Center for Modeling Biological Systems and Data Analysis, Department of Functional Science, Victor Babes University of Medicine and Pharmacy Timisoara, 300041 Timisoara, Romania

**Keywords:** ERCP, propofol sedation, adverse events, anaesthesia, dose–response, patient safety, ASA score, pulmonary disease

## Abstract

**Background/Objectives:** Endoscopic retrograde cholangiopancreatography (ERCP) requires deep sedation, which is increasingly provided by anaesthetists using propofol-based regimens. However, real-world data on the incidence and predictors of anaesthesia-related adverse events (AEs) in this setting remain limited. The objective of this study was to assess the frequency, predictors, and clinical significance of adverse events during anaesthetist-delivered sedation for ERCP, based on a propofol regimen. **Methods:** We conducted a retrospective single-centre cohort study including 388 consecutive adult patients who underwent ERCP with propofol-based sedation administered by an anaesthetist. Adverse events were classified into three tiers: Tier 1 (any adverse physiological events, including haemodynamic and respiratory threshold crossings), Tier 2 (clinically significant events requiring pharmacological intervention—the primary regression outcome), and Tier 3 (high-severity outcomes, reported descriptively). Independent predictors of Tier 2 events were identified using multivariable logistic regression. **Results:** Adverse physiological events occurred in 220 patients (56.7%), the majority of which were minor and self-limiting. Clinically significant events requiring active pharmacological intervention occurred in 108 patients (27.8%), with vasopressor-treated hypotension as the predominant component (88 patients, 22.7%). All bradycardia episodes required atropine administration (34 patients, 8.8%), while desaturation was largely self-limiting, with advanced airway management required in only three patients (0.8%). High-severity outcomes were rare (9 patients, 2.3%). In multivariable logistic regression predicting clinically significant adverse events, propofol dose (OR 1.20 per 10 mg, 95% CI 1.14–1.25, *p* < 0.001), ASA physical status (OR 1.63, 95% CI 1.07–2.49, *p* = 0.024), age (OR 1.04 per year, 95% CI 1.01–1.07, *p* = 0.007), and ketamine use, confounded by indication (OR 2.18, 95% CI 1.14–4.14, *p* = 0.018) were independent predictors. Model fit was good (Nagelkerke R^2^ = 0.43). **Conclusions:** Adverse events are frequent when defined using inclusive criteria, but are predominantly minor in severity. Propofol dose is the principal modifiable risk factor, demonstrating a consistent dose–response relationship across multiple adverse outcomes. ASA physical status and age further identify patients at increased risk of clinically significant events requiring intervention. Ketamine use was associated with increased odds of adverse events; however, this association is likely confounded by indication and should not be interpreted as a direct causal effect. These findings support stepwise propofol titration guided by clinical sedation assessment, with avoidance of anticipatory dosing particularly in older patients and those with higher ASA scores, and highlight the safety of anaesthetist-led sedation in this setting.

## 1. Introduction

Endoscopic retrograde cholangiopancreatography (ERCP) is a complex gastrointestinal endoscopic procedure used to diagnose and treat biliary and pancreatic pathology. Its complexity leads to prolonged duration and the requirement for deep sedation and immobility making anaesthesia a cornerstone of procedural success [1,2].

The delivery of sedation for ERCP has evolved considerably over the past two decades. Where conscious sedation administered by the endoscopist was once standard, there has been a progressive shift towards deeper, anaesthetist-delivered sedation, predominantly based on propofol. This transition reflects both the increasing procedural complexity of modern ERCP, including sphincterotomy, stenting, and lithotripsy, and the growing recognition that deeper sedation improves procedural conditions and patient tolerance [3]. However, anaesthetist-delivered propofol sedation is not without risk. The narrow therapeutic window of propofol, combined with the physiological vulnerability of patients typically referred for ERCP-older age, multiple comorbidities, compromised cardiorespiratory reserve, creates conditions in which hemodynamic and respiratory adverse events are frequent [4].

Despite the widespread adoption of this practice, the literature on the safety profile of anaesthetist-delivered sedation specifically for ERCP remains limited, with most available data either pooled across multiple endoscopic procedures or derived from settings where sedation is administered by non-anaesthesiologist personnel. In clinical practice, propofol is rarely used as a single sedative, and it is frequently combined with opioids such as fentanyl for analgesia, benzodiazepines such as midazolam for anxiolysis and amnesia, and, in selected cases, low-dose ketamine as an adjunct to reduce total propofol requirements or to manage procedurally complex patients. The independent contribution of each agent to the overall adverse event profile, and in particular the interactions between adjunct drug use and propofol dose–response relationships, have not been systematically described in this procedural context [5,6]. Furthermore, the incidence and clinical profile of high-severity outcomes, including the need for sedation reversal, advanced airway management, intensive care admission, and mortality, remain poorly defined in cohorts under anaesthetist supervision.

The present study aimed to describe the incidence of complications and identify independent predictors of anaesthesia-related adverse events in a consecutive cohort of 388 patients undergoing ERCP under anaesthetist-delivered propofol-based sedation, and to characterise the clinical profile of high-severity outcomes in this population. Specifically, we examined the dose–response relationship between propofol and individual adverse events, the contribution of patient-related risk factors, including ASA physical status, pulmonary disease, and concomitant ketamine use, and the overall safety margin of this sedation model in a real-world tertiary centre setting.

## 2. Materials and Methods

### 2.1. Study Design and Patient Population

This was a retrospective, single-centre cohort study conducted at “Pius Brînzeu” Emergency County Hospital in Timișoara, Romania. All consecutive adult patients who underwent ERCP under anaesthetist-delivered sedation between February 2025 and February 2026 were considered for inclusion. The requirement for individual informed consent was waived given the retrospective observational nature of the study.

Inclusion criteria: age ≥ 18 years; ERCP performed under anaesthetist-delivered sedation; complete anaesthetic, recovery, and procedural records available; cases where propofol was the primary sedative agent; available baseline clinical data; ASA I-IV; sedation intended as the primary initial technique.

Exclusion criteria: paediatric patients; procedures performed under general anaesthesia with endotracheal intubation from the outset; pre-existing endotracheal intubation or mechanical ventilation; severe baseline hemodynamic instability; baseline oxygen dependence; documented acute altered mental status before the procedure; known allergy or contraindication to propofol or adjunct agents; pregnancy; incomplete records; refusal of data use.

**Figure 1:**
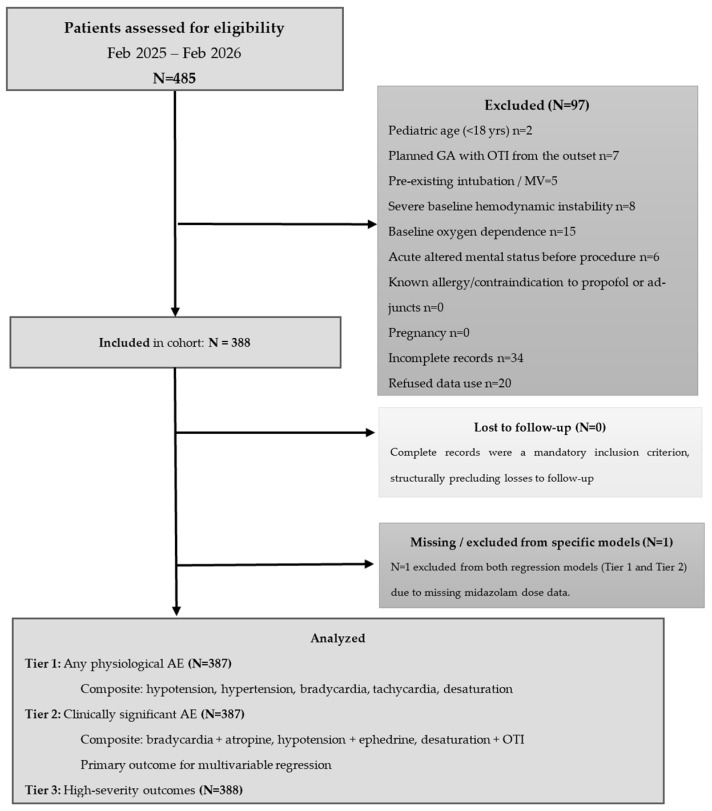
STROBE participant flow diagram. Consecutive adult patients undergoing ERCP with propofol-based anaesthetist-delivered sedation were assessed for eligibility between February 2025 and February 2026. Patients meeting any exclusion criterion were excluded prior to enrolment. The final cohort of 388 patients was analysed across three outcome tiers: Tier 1 (any adverse physiological events, *n* = 387), Tier 2 (clinically significant adverse event requiring pharmacological intervention, *n* = 387, primary regression outcome), and Tier 3 (high-severity outcomes, *n* = 388, descriptive). One patient with missing outcome data was excluded from the Tier 1 and 2 regression models. ERCP = endoscopic retrograde cholangiopancreatography; AE = adverse event; OTI = endotracheal intubation; GA = general anaesthesia; MV = mechanical ventilation.

A total of 388 patients were included in the final analysis. The full STROBE selection sequence is presented in Figure 1.

### 2.2. Sedation Protocol

All procedures were performed in a dedicated ERCP unit equipped with a full anaesthesia workstation and standard monitoring capabilities. Upon arrival, patients were positioned and connected to standard monitoring, including non-invasive blood pressure (NIBP), pulse oximetry (SpO_2_), and continuous electrocardiography (ECG). Monitoring was performed using a GE CARESCAPE B650 (GE Healthcare, Helsinki, Finland) anaesthesia monitoring system.

Peripheral intravenous access was established in all patients using an 18G or 20G cannula. Baseline physiological parameters, including heart rate (HR), systolic blood pressure (SBP), and SpO_2_, were recorded before sedation. Supplemental oxygen was administered to all patients via nasal cannula at a flow rate of 4 L/min following baseline SpO_2_ assessment.

All procedures were conducted under anaesthetist-delivered sedation. Before the procedure, patients were assessed for adequate fasting in accordance with institutional guidelines and for potential airway difficulty as part of the pre-anaesthetic evaluation. A crystalloid infusion (Plasmalyte, Baxter Healthcare, Lessines, Belgium) was initiated at a dose of approximately 10 mL/kg and maintained throughout the procedure. Pre-anaesthetic medication consisted of midazolam (Midazolam Hypericum, 5 mg/mL, Laboratoire Aguettant, Lyon, France), administered as an intravenous bolus (10–20 µg/kg), in the absence of contraindications. Sedation was induced using a combination of fentanyl 0.5–1 µg/kg (Fentanyl Kalceks, 50 mcg/mL, Akciju sabiedrība Kalceks, Riga, Latvia) and propofol 0.5 mg/kg (Propofol MCT/LCT, 10 mg/mL, Fresenius Kabi, Bad Homburg, Germany), titrated to clinical effect. Maintenance of sedation was achieved with intermittent bolus administration of propofol (0.15–0.25 mg/kg), guided by clinical assessment and target sedation depth. Additional fentanyl boluses were administered when required for analgesia, based on indirect indicators of nociception, including increases in heart rate and systolic blood pressure. In selected cases, low-dose ketamine (Ketamine, Calypsol 50 mg/mL, Gedeon Richter Ltd., Budapest, Hungary) was administered at the discretion of the attending anaesthetist as an adjunct to the propofol-based regimen, defined as a sub-dissociative intravenous bolus of 0.1–0.3 mg/kg, providing analgesia while preserving airway reflexes and haemodynamic stability. Ketamine was reserved for patients in whom additional analgesia was required due to procedural complexity, or in whom haemodynamic instability was anticipated, serving as an adjunct to reduce total propofol requirements. A 500 µg atropine (sulfat de atropina, 1 mg/mL, Takeda, Berlin, Germany) bolus was used for the management of significant intraoperative bradycardia, and a 10 mg ephedrine (ephedrini hydrochloridum, 50 mg/mL, Zentiva, Bucharest, Romania) bolus in cases of significant hypotension.

Depth of sedation was assessed using the Modified Observer’s Assessment of Alertness/Sedation (MOAA/S) scale, dosing was adjusted to maintain the target level of sedation (MOAA/S 3), with transient deeper levels of sedation (MOAA/S ≤ 2) permitted during periods of increased procedural stimulation in order to ensure optimal procedural conditions. Intra-procedural monitoring included NIBP measurements at 5 min intervals, with continuous ECG and pulse oximetry monitoring. Heart rate and SpO_2_ values were recorded at 5 min intervals or more frequently in the presence of clinically significant deviations, including desaturation, bradycardia, or tachycardia. Post-procedural recovery was assessed using the Aldrete score at predefined intervals, and patients were monitored until adequate recovery criteria were met.

### 2.3. Data Collection and Variable Definitions

Demographic and clinical data were retrospectively extracted from anaesthetic and procedural records and entered into a structured database. Variables collected included: age, sex, ASA physical status score, presence of chronic pulmonary disease, chronic hypertension, ACE inhibitor use, anticoagulant and antiplatelet therapy, and statin use. Procedural variables included procedure duration, diagnosis (benign vs. malignant), presence of angiocholitis, stenting performed, number of cannulation attempts, Wirsung cannulations, and time from hospital admission to procedure. Anaesthetic variables included doses of propofol, fentanyl, midazolam, ketamine, atropine, and ephedrine; baseline and intraoperative heart rate and systolic blood pressure; baseline and minimum intraoperative SpO_2_; and MOAA/S scores at baseline and at 3 min. Recovery was assessed using the Aldrete score at 0, 5, and 15 min post-procedure. Adverse events were recorded from administration of the first sedative agent to discharge from the recovery area. Hypotension was defined as SBP < 90 mmHg or a decrease >20% from baseline; hypertension as SBP > 160 mmHg or an increase >20% from baseline; bradycardia as HR < 50 bpm or a decrease >20% from baseline; tachycardia as HR > 100 bpm or an increase >20% from baseline; and desaturation as SpO_2_ < 90% or a decrease >3% from baseline. Classification was triggered if either criterion was met.

#### 2.3.1. Adverse Physiological Events

Five intraoperative adverse physiological events were defined and recorded by the attending anaesthetist: bradycardia, tachycardia, arterial hypotension, arterial hypertension, and oxygen desaturation. Bradycardia was defined as a heart rate below 50 beats per minute or a reduction of more than 20% from the individual patient’s baseline value. Tachycardia was defined as a heart rate exceeding 100 beats per minute. Arterial hypotension was defined as any episode recorded by the attending anaesthetist reflecting either an absolute systolic blood pressure below 90 mmHg or a clinically significant relative decrease from the individual patient’s baseline; the latter criterion was applied in hypertensive patients in whom a proportional drop carried haemodynamic significance despite remaining above the absolute threshold. Arterial hypertension was defined as a systolic blood pressure exceeding 160 mmHg or an increase of more than 20% from baseline. Oxygen desaturation was defined as a peripheral oxygen saturation below 90% on continuous pulse oximetry.

For bradycardia and hypotension, the number of episodes was recorded continuously. For desaturation, tachycardia, and hypertension, occurrence was recorded as a binary variable. Hypotension severity was further characterised by total ephedrine dose administered, which reflects cumulative vasopressor burden and serves as a proxy for episode duration and haemodynamic significance in the absence of continuous blood pressure waveform data. Continuous severity metrics—minimum heart rate, minimum systolic blood pressure, maximum heart rate, maximum systolic blood pressure, and minimum SpO_2_—were recorded for all patients throughout the observation window.

#### 2.3.2. Clinical Intervention Variables

Clinical interventions were recorded separately from physiological events to permit independent assessment of event severity. Atropine was administered for significant bradycardia at a bolus dose of 500 µg (sulfat de atropina 1 mg/mL, Takeda, Berlin, Germany); total dose administered was recorded. Ephedrine was administered for significant hypotension at a bolus dose of 10 mg (ephedrini hydrochloridum 50 mg/mL, Zentiva, Bucharest, Romania); total dose administered was recorded. Advanced airway management (endotracheal intubation) was recorded in cases of refractory or severe desaturation not responsive to conservative measures. For the purposes of regression modelling, propofol dose was rescaled to a per-10 mg increment. Urgency of the procedure was classified based on time from hospital admission to procedure: urgent (≤24 h), semi-urgent (25–47 h), and elective (≥48 h).

#### 2.3.3. Outcome Classification

Three outcome tiers were defined for analysis. Tier 1 (any physiological AE) was defined as the occurrence of one or more of the five physiological events described above, and corresponds to the overall rate of anaesthetic adverse events. Tier 2 (clinically significant AE) was defined as the co-occurrence of a physiological event and its corresponding pharmacological intervention: bradycardia treated with atropine, hypotension treated with ephedrine, or desaturation requiring endotracheal intubation. This tier was used as the primary outcome for multivariable regression analysis, as it reflects events requiring active clinical management rather than transient, self-limiting physiological excursions. Tier 3 (high-severity outcomes) comprised the following: need for sedation reversal with flumazenil, procedure interruption due to adverse events, advanced airway management, ICU admission, death within 24 h, and in-hospital death. Tier 3 outcomes were analysed descriptively given their low expected frequency.

A separate procedural adverse event composite was defined as the occurrence of any of the following: purulent bile, procedural bleeding, or perforation.

### 2.4. Statistical Analysis

Statistical analyses were performed using Python 3.12 (pandas 2.2, scipy 1.13, statsmodels 0.14, scikit-learn 1.5). Normality of continuous variables was assessed using the Shapiro–Wilk test. Continuous variables are reported as median and interquartile range (IQR); categorical variables as frequency and percentage. Group comparisons used the Mann–Whitney U test or Kruskal–Wallis test for continuous variables and Fisher’s exact test or chi-square test for categorical variables, as appropriate.

Univariate logistic regression was performed for each candidate predictor against the Tier 2 clinically significant AE outcome. A core set of clinically pre-specified predictors: age, sex, ASA physical status, propofol dose, procedure duration, and pulmonary disease was included in all multivariable models regardless of univariate significance, on the basis of established clinical relevance. Additional candidate variables with *p* < 0.1 in univariate analysis were considered for model inclusion on an outcome-specific basis.

The multivariable logistic regression model predicting Tier 2 AE included the following pre-specified predictors: age, sex, ASA physical status, propofol dose (per 10 mg increment), ketamine use (binary), midazolam dose, procedure duration, pulmonary disease, arterial hypertension as a comorbidity, and malignant diagnosis.

Fentanyl was administered to a small subset of patients and exhibited complete separation in both univariate and multivariable analyses, precluding stable coefficient estimation regardless of coding strategy; it was therefore excluded from the regression model and is reported descriptively.

Multicollinearity was assessed using the variance inflation factor (VIF); variables with VIF > 5 were excluded. Model discrimination was assessed by the area under the receiver operating characteristic curve (AUC). Calibration was assessed using the Hosmer–Lemeshow test. Model fit was quantified using McFadden’s and Nagelkerke’s pseudo-R^2^. For comparability, the same predictor set was applied in a parallel model predicting Tier 1 (any AE), and results are presented alongside the Tier 2 model to illustrate the differential predictive signal captured by each outcome definition. Odds ratios (OR) are reported with 95% confidence intervals. A two-sided *p*-value < 0.05 was considered statistically significant throughout.

High-severity outcomes were described using absolute frequencies and rates only; formal predictive modelling was not performed for these outcomes due to the low number of events (fewer than five events per potential predictor for most outcomes).

### 2.5. Ethics

This study was conducted in accordance with the Declaration of Helsinki. Ethical approval was granted by Ethics Committee of the Doctoral School, “Victor Babeș” University of Medicine and Pharmacy Timișoara (UMFT), Romania, approval number 15. Individual patient consent was waived given the retrospective design and de-identified nature of the data.

### 2.6. Generative AI Usage Statement

During the preparation of this manuscript, AI tools were used to assist with language editing and code development. All outputs were critically reviewed by the authors, and all scientific content remains the responsibility of the authors.

## 3. Results

### 3.1. Patient Characteristics

A total of 388 patients were included in the final analysis. The cohort was predominantly older, with a median age of 70 years (IQR 58.8–77.0), and 57.7% were female. ASA physical status score was median 3 (IQR 2–3), reflecting a population with substantial comorbidity burden. Chronic hypertension was present in 60.8% of patients, pulmonary disease in 15.7%, and ACE inhibitor use in 39.7%. Malignant diagnosis was the indication for ERCP in 66.5% of cases, and angiocholitis was documented in 44.7%.

Median procedure duration was 30 min (IQR 20–40). Median propofol dose was 150 mg (IQR 100–200). Midazolam was used in 63.1% of patients (median dose 1 mg, IQR 0–2), ketamine in 26.0% (*n* = 101; median dose among recipients 20 mg, IQR 20–30), and fentanyl in virtually all patients (median dose 0.1 mg). Detailed patient characteristics are presented in Table 1.

Ketamine and midazolam doses reported for the full cohort, including non-recipients. Median dose among ketamine recipients (*n* = 101): 20 mg (IQR 20–30). Patients who received ketamine were significantly younger (median 66 vs. 71 years, *p* = 0.011), had lower baseline systolic blood pressure (129 vs. 135 mmHg, *p* = 0.012), and higher rates of anaesthetic adverse events (66.3% vs. 53.3%, *p* = 0.026) compared to those who did not receive ketamine.

The distribution of adverse physiological events recorded during the observation window is presented in Table 2, with patient flow illustrated in Figure 1.

### 3.2. Anaesthetic Adverse Events

**Figure 2:**
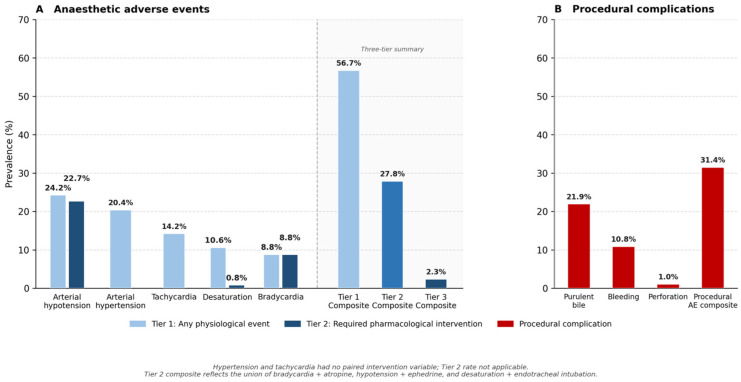
Adverse event overview by outcome tier. (**A**) Individual physiological event rates and three-tier composite summary (*n*=388). Light blue = Tier 1 (any physiological event); dark blue = Tier 2 (events requiring pharmacological intervention). The Tier 2 hypotension rate (22.7%) reflects ephedrine-treated episodes only; hypertension and tachycardia had no paired intervention variable and are excluded from the Tier 2 composite. (**B**) Procedural complication rates. The procedural AE composite (31.4%) reflects the union of purulent bile, bleeding, and perforation and is not additive. AE = adverse event.

Adverse physiological events (Figure 2A), constituting Tier 1 of the outcome frame-work, occurred in 220 patients (56.7%). The distribution across event types is presented in Table 2. The most frequent events were arterial hypotension (94 patients, 24.2%; median minimum SBP 80 mmHg, IQR 76–88), tachycardia (55 patients, 14.2%; median maximum HR 108 bpm, IQR 103.5–110), and hypertension (79 patients, 20.4%; median maximum SBP 164 mmHg, IQR 156–171). Desaturation occurred in 41 patients (10.6%; median minimum SpO_2_ 88%, IQR 86–89) and bradycardia in 34 patients (8.8%; median minimum HR 42 bpm, IQR 40–44).

Clinically significant adverse events (Figure 2B) requiring pharmacological intervention (Tier 2) occurred in 108 patients (27.8%). The predominant component was vasopressor-treated hypotension (88 patients, 22.7%; median ephedrine dose 20 mg). All 34 patients with documented bradycardia required atropine administration (100%; median dose 0.50 mg, IQR 0.50–0.88), indicating that no episode of bradycardia was self-limiting in this cohort. Desaturation, by contrast, was largely self-resolving: of 41 patients with documented desaturation, only three (7.3%) required endotracheal intubation, with the remainder managed conservatively through supplemental oxygen adjustment and airway positioning. Fourteen patients (13.0%) met the criteria for more than one Tier 2 component simultaneously.

High-severity outcomes (Tier 3) occurred in nine patients (2.3%), confirming the overall safety margin of anaesthetist-delivered propofol sedation in this cohort.

In univariate analysis, propofol dose, procedure duration, fentanyl dose, ASA score, ketamine use, and MOAA/S minimum were significantly associated with the anaesthetic adverse event composite (all *p* < 0.05). Age, sex, pulmonary disease, ACE inhibitor use, hypertension, and midazolam use were not significant univariate predictors. Full univariate results are presented in Table 3.

**Figure 3:**
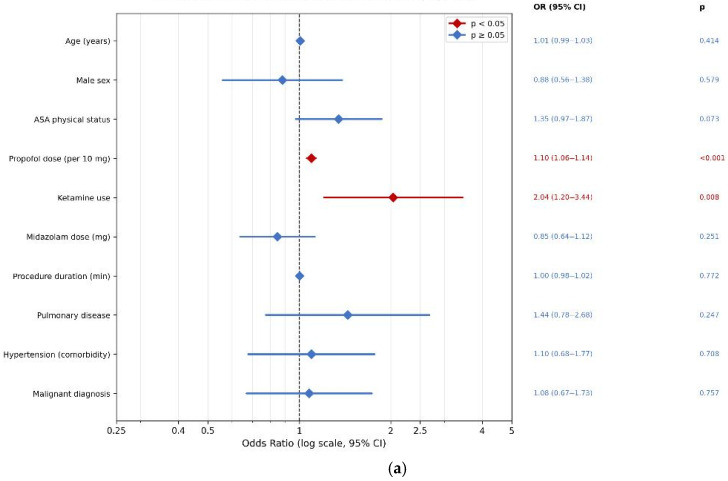

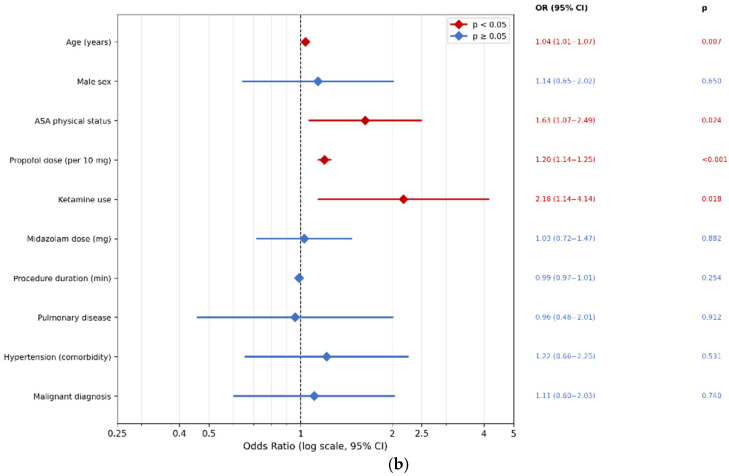
Logistic regression for anaesthetic adverse events: (**a**) Tier 1 model—predictors of any adverse physiological events; (**b**) Tier 2 model—predictors of clinically significant adverse events requiring intervention.

In multivariable logistic regression predicting clinically significant adverse events (Tier 2), propofol dose was the strongest independent predictor (OR 1.20 per 10 mg, 95% CI 1.14–1.25, *p* < 0.001), confirming a dose–response relationship consistent across all pharmacological intervention endpoints. ASA physical status (OR 1.63, 95% CI 1.07–2.49, *p* = 0.024), age (OR 1.04 per year, 95% CI 1.01–1.07, *p* = 0.007), and ketamine use (OR 2.18, 95% CI 1.14–4.14, *p* = 0.018) were also independently associated with clinically significant events. Sex, midazolam dose, procedure duration, pulmonary disease, arterial hypertension as a comorbidity, and malignant diagnosis were not independently associated with Tier 2 outcomes. Model fit was good (Nagelkerke R^2^ = 0.43, McFadden R^2^ = 0.30), representing a substantial improvement over the parallel Tier 1 model (Nagelkerke R^2^ = 0.20), reflecting the superior outcome specification of the intervention-defined endpoint. Results are presented in Table 4, with corresponding forest plots in Figure 3a (Tier 1) and Figure 3b (Tier 2).

### 3.3. Predictors of Individual Adverse Events

Given the clinical relevance of each adverse event type, separate multivariate models were built for bradycardia, hypotension, and desaturation: the three outcomes with sufficient event counts for modelling (*n* = 34, 94, and 41 respectively). Tachycardia and hypertension models showed poor explanatory power (Nagelkerke R^2^ = 0.036 and 0.053 respectively) and are reported descriptively.

**Figure 4:**
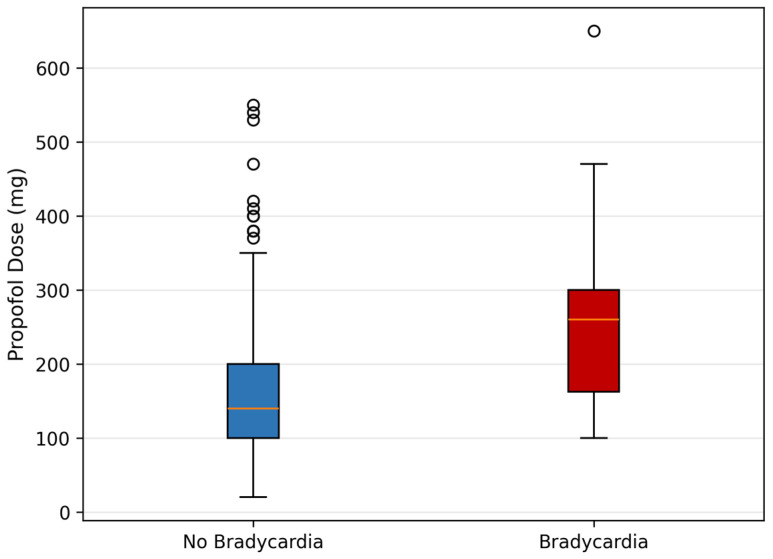
Propofol dose distribution by bradycardia status. Box boundaries represent the interquartile range, the horizontal line indicates the median, whiskers extend to 1.5 × IQR, and circles represent individual outliers. No Bradycardia *n* = 354, Bradycardia *n* = 34. Mann-Whitney U test, *p* < 0.001.

Bradycardia was strongly and independently associated with propofol dose (OR 1.08 per 10 mg, 95% CI 1.05–1.12, *p* < 0.001), with no other predictor reaching significance. Patients who developed bradycardia had received substantially higher propofol doses than those who did not (median 260 mg, IQR 162.5–300, vs. 140 mg, IQR 100–200; *p* < 0.001), confirming a clear dose–response relationship. The bradycardia model showed moderate fit (Nagelkerke R^2^ = 0.197). The dose–response relationship between propofol and bradycardia is illustrated in Figure 4.

Hypotension was independently predicted by propofol dose (OR 1.16 per 10 mg, 95% CI 1.11–1.21, *p* < 0.001) and ASA score (OR 1.77, 95% CI 1.16–2.69, *p* = 0.008), indicating that both anaesthetic exposure and baseline patient frailty contribute independently to hemodynamic compromise. The hypotension model showed good explanatory power (Nagelkerke R^2^ = 0.375). Among patients requiring vasopressor support, the median total ephedrine dose was 20 mg, (IQR [10–30]), with seven patients requiring more than 30 mg, indicating sustained or recurrent hypotension beyond a single self-limiting episode.

Desaturation was independently predicted by propofol dose (OR 1.10 per 10 mg, 95% CI 1.05–1.14, *p* < 0.001), ASA score (OR 1.70, 95% CI 1.00–2.90, *p* = 0.049), and pulmonary disease (OR 2.47, 95% CI 1.06–5.76, *p* = 0.036). The desaturation model showed moderate fit (Nagelkerke R^2^ = 0.271).

ACE inhibitor use was not a significant predictor of bradycardia (OR 0.84, *p* = 0.687), hypotension (OR 1.01, *p* = 0.970), or desaturation (OR 1.07, *p* = 0.866) in any of the multivariate models.

Multivariate results for all three outcomes are presented in Table 5.

### 3.4. Propofol Dosing and ASA Score

**Figure 5:**
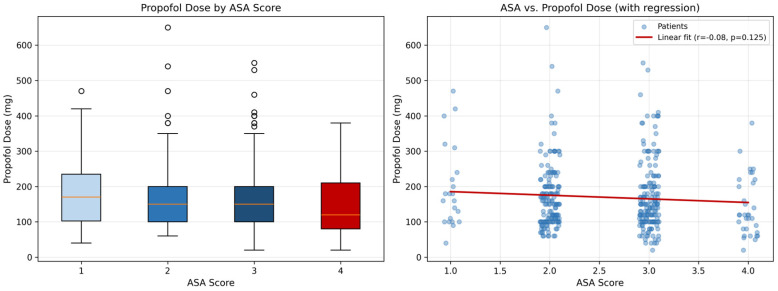
ASA and propofol dose relationship. (**Left**) Box plot by ASA group (*n* = 388); box = IQR, line = median, whiskers = 1.5 × IQR, circles = outliers. (**Right**) Individual patient doses with linear regression overlay (r = −0.08, *p* = 0.125).

Propofol doses did not differ significantly across ASA groups (median: ASA 1—170 mg, ASA 2—150 mg, ASA 3—150 mg, ASA 4—120 mg; Kruskal–Wallis *p* = 0.216), suggesting that anaesthetists titrated propofol to clinical effect rather than adjusting proactively for comorbidity severity. The interquartile ranges were similar between ASA II and III patients and remained wide overall, reflecting considerable inter-individual variability in dosing (Figure 5).

### 3.5. Atropine Use

Atropine was administered in 34 patients (8.8%), at a median dose of 0.50 mg (IQR 0.50–0.88; mean 0.71 ± 0.41 mg; range 0.50–2.00 mg). All 34 patients who received atropine had documented intraoperative bradycardia. Patients who required atropine had received higher propofol doses than those who did not.

### 3.6. High-Severity Outcomes

High-severity outcomes were rare across the cohort. Flumazenil was required in five patients (1.29%), procedure interruption occurred in two (0.52%), advanced airway management (endotracheal intubation) was necessary in three (0.77%), ICU admission in four (1.03%), death within 24 h in two (0.52%), and in-hospital death in two (0.52%).

All high-severity outcomes occurred in patients who had also experienced at least one anaesthetic adverse event in the composite, though the association did not reach statistical significance given the low event counts (Table 6).

Patients requiring flumazenil reversal were characterised by high propofol exposure, universal benzodiazepine co-administration, and frequent anaesthetic adverse events, suggesting a profile of cumulative sedative burden. All patients received propofol-based sedation (propofol doses ≥ 120 mg) in combination with midazolam and fentanyl. Out of the five patients in the cohort requiring reversal of benzodiazepine, four had ASA score ≥ 3, with one patient demonstrating significant desaturation (minimum SpO_2_ 85%). In regard to recovery, lower initial Aldrete scores were observed in all patients requiring reversal.

All three patients requiring endotracheal intubation shared a profile of advanced comorbidity (ASA physical status scores were 3 and 4), high anaesthetic exposure, and pre-existing pulmonary disease. Propofol doses were 300 mg, 270 mg, and 250 mg—all substantially above the cohort median of 150 mg. All three patients developed desaturation refractory to supplemental oxygen adjustment and airway repositioning, prompting elective endotracheal intubation by the attending anaesthetist. Two patients were successfully extubated in the recovery area without further complications. The third patient was transferred to the ICU and extubated three hours later following stabilisation of concurrent haemodynamic instability; this patient did not require ICU admission solely on the basis of the airway event.

Both in-hospital deaths occurred in patients with advanced disease and high procedural complexity. One patient had ASA score 3 with a malignant diagnosis complicated by angiocholitis; the other had ASA score 4 with purulent bile drainage at ERCP. Both patients experienced Tier 2 anaesthetic adverse events during the procedure—specifically bradycardia and hypotension—and one additionally developed desaturation. Death occurred within 24 h in both cases. Cause of death was sepsis in one patient and disease progression with multi-organ failure in the other. Neither death was directly attributable to the anaesthetic technique based on available records.

## 4. Discussion

This study examined the incidence and predictors of anaesthesia-related adverse events in a consecutive cohort of 388 patients undergoing ERCP under anaesthetist-delivered propofol-based sedation. Three principal findings emerge. First, while adverse physiological events were common, affecting 56.7% of patients, the majority were transient and self-limiting; clinically significant events requiring pharmacological intervention occurred in 27.8% of patients, and high-severity outcomes were rare (2.3%). This distinction is clinically important: a composite adverse event rate that conflates minor haemodynamic fluctuations with events requiring active management risks misrepresenting the safety profile of a procedure in which some degree of physiological variation is expected and manageable. Second, propofol dose was the most consistent independent predictor across all outcome models, with a dose–response relationship that persisted after adjustment for patient-level risk factors. Third, patient-level characteristics—age and ASA physical status—emerged as significant independent predictors of clinically significant events but not of any physiological excursion, suggesting that these factors determine which patients will require intervention, rather than merely which patients will experience a measurable physiological deviation. This differential signal, visible only when the outcome is defined by intervention receipt, underscores the methodological importance of distinguishing between physiological events and clinically significant adverse events in sedation safety research.

### 4.1. Frequency and Nature of Adverse Events

The composite anaesthetic adverse event rate of 56.7% observed in our cohort is higher than figures reported in studies of endoscopist-administered sedation, which typically describe adverse event rates in the range of 10 to 30% [7]. This discrepancy is largely attributable to differences in outcome definition: our composite included hemodynamic instability requiring intervention, such as a single episode of hypotension or tachycardia, as well as the use of atropine or ephedrine for pharmacological management of these adverse events. When defined in this inclusive manner, the observed rate is consistent with reports of anaesthetist-delivered propofol sedation for advanced endoscopic procedures that apply similarly broad composite definitions [8]. It also reflects the clinical reality of deep procedural sedation in a population with a median ASA score of 3, where hemodynamic reactivity to propofol is expected and pharmacological management is routine rather than exceptional. Sedation was delivered by anaesthetists in training with at least three years of experience in anaesthesia and intensive care, under direct supervision, reflecting routine staffing at our centre. Importantly, despite this real-world setting, high-severity outcomes remained rare. Current guidelines emphasise that the safety of procedural sedation depends on appropriate training, competence, and monitoring rather than provider grade alone, provided that clinicians are adequately skilled in airway management and hemodynamic support [9].

The predominance of haemodynamic over respiratory adverse events is consistent with the known pharmacodynamic profile of propofol, which produces dose-dependent vasodilation and cardiac depression at procedural sedation doses [10]. The relatively low desaturation rate (10.6%) likely reflects routine supplemental oxygen use, which attenuates but does not eliminate hypoxemic episodes in patients with compromised respiratory reserve [11].

The 100% concordance between bradycardia and atropine administration confirms that no bradycardic episode was self-limiting in this cohort, distinguishing it from the largely self-resolving pattern of desaturation.

### 4.2. Propofol as the Central Dose-Dependent Risk Factor

The consistent identification of propofol dose as an independent predictor across all three individual adverse event models—bradycardia (OR 1.08 per 10 mg), hypotension (OR 1.16 per 10 mg), and desaturation (OR 1.10 per 10 mg)—reinforces the established dose–response relationship between propofol exposure and cardiorespiratory depression [12]. The magnitude of the effect is clinically meaningful: a 50 mg increment in propofol dose, which is well within the range of routine titration, corresponds to approximately a 50% increase in the odds of bradycardia and a near-doubling of the odds of hypotension. Patients who developed bradycardia received a median propofol dose of 260 mg compared to 140 mg in those who did not, a difference that is both statistically robust and clinically actionable.

These findings argue for a titration-first approach to propofol dosing in ERCP sedation. The median dose in our cohort of 150 mg over a 30 min procedure reflects a conservative regimen by published standards; nevertheless, the dose–response relationship observed (OR 1.20 per 10 mg for clinically significant adverse events) indicates that even within this range, incremental dose reductions carry measurable safety benefits. We do not propose a specific ceiling dose, as the minimum effective dose is necessarily individual and procedurally dependent, being determined by patient responsiveness, procedural stimulation intensity, and target sedation depth. The clinically actionable recommendation is therefore not a fixed dose threshold but a titration discipline: stepwise dose adjustment guided by MOAA/S assessment, with avoidance of anticipatory boluses in the absence of clinical indication, particularly in patients with ASA score ≥3 or advanced age. [13].

### 4.3. The Role of Adjunct Agents

The independent association of ketamine use with the anaesthetic adverse event composite (OR 2.04) requires careful interpretation. Ketamine is typically added to propofol regimens to reduce total propofol requirements through synergistic sedation and to provide procedural analgesia in complex cases [14]. In our cohort, patients who received ketamine were younger, had lower baseline blood pressure, and had a higher composite adverse event rate, a pattern more consistent with selection for perceived procedural or hemodynamic complexity than with a direct pharmacological harm effect of ketamine itself. The dissociative and sympathomimetic properties of ketamine would in theory be protective against hypotension and bradycardia, and this is reflected in the non-significant association between ketamine and these specific outcomes in the multivariate models.

Fentanyl was excluded from the multivariable model due to complete separation, precluding stable coefficient estimation; its descriptive role in the sedation protocol is noted in Section 2.2.

### 4.4. Patient-Related Risk Factors: ASA Score and Pulmonary Disease

ASA score emerged as an independent predictor of both hypotension (OR 1.77) and desaturation (OR 1.70), confirming that baseline physiological reserve modifies the cardiorespiratory response to propofol in a clinically relevant manner. This finding is consistent with the broader anaesthetic literature and supports the use of ASA score as a risk stratification tool prior to procedural sedation, even in settings where a full anaesthetic workup is not performed [15].

Pulmonary disease independently doubled the odds of desaturation (OR 2.47), a finding with direct clinical implications for patient preparation and monitoring. Patients with pre-existing respiratory compromise—chronic obstructive pulmonary disease, asthma, or other pulmonary conditions—represent a subgroup in which supplemental oxygen delivery, careful propofol titration, and heightened vigilance for respiratory depression are warranted [16,17].

### 4.5. ACE Inhibitor Use and Propofol-Induced Hypotension

ACE inhibitor use, present in 39.7% of our cohort, was not associated with anaesthetic adverse events in univariate analysis (*p* = 0.278) or in any of the multivariable models. This is a clinically relevant negative finding. The theoretical concern that chronic renin-angiotensin system blockade potentiates propofol-induced hypotension through enhanced nitric oxide-mediated vasodilation [18] has informed preoperative withholding practices in some centres. Our data do not support this practice in the context of propofol-based procedural sedation for ERCP, and are consistent with recent randomised evidence suggesting that continuation of ACE inhibitors does not increase haemodynamic instability at the doses used for procedural sedation [19]. Routine preoperative withholding of ACE inhibitors prior to ERCP sedation does not appear warranted based on these findings.

### 4.6. High-Severity Outcomes and the Safety Margin of Anaesthetist-Delivered Sedation

The low incidence of high-severity outcomes—flumazenil 1.29%, endotracheal intubation 0.77%, ICU admission 1.03, and in-hospital mortality 0.52%—is the most clinically important finding of this study. In a cohort characterised by advanced age, high comorbidity burden, and a majority of malignant indications, these rates compare favourably with published benchmarks for ERCP sedation across all provider models [20]. Notably, all high-severity outcomes occurred in patients who had also experienced at least one component of the anaesthetic adverse event composite, suggesting a continuum of severity rather than unpredictable catastrophic events.

The mortality rate of 0.52% requires contextualisation. Both deaths occurred in patients with advanced malignant disease, ASA score 3 or higher, and significant procedural complexity, and neither death was directly attributable to the anaesthetic technique based on available records. This pattern is consistent with the reported 30-day mortality in ERCP populations with comparable comorbidity profiles, where procedure-related and disease-related mortality are difficult to disentangle [21].

The availability of an anaesthetist throughout the procedure is likely a contributing factor to the containment of adverse events within manageable severity in our cohort. The capacity for immediate pharmacological reversal, airway rescue, and hemodynamic resuscitation—all of which were required in small numbers of patients—is inherent to anaesthetist-led models and represents a structural safety advantage over nurse-administered or endoscopist-directed sedation, particularly for the high-risk ERCP population [22,23,24].

### 4.7. Limitations

Several limitations of this study should be acknowledged. The retrospective single-centre design limits generalizability and introduces the possibility of unmeasured confounding. The inclusive definition of the composite anaesthetic adverse event, while clinically justified, inflates the apparent adverse event rate compared to studies using more restrictive outcome definitions, and direct comparisons with other cohorts should account for this methodological difference. The low incidence of high-severity outcomes, while reassuring, precluded formal predictive modelling for these endpoints, and the study was not powered to detect predictors of rare outcomes such as endotracheal intubation or ICU admission. The absence of continuous hemodynamic monitoring data limited our ability to characterise the duration and trajectory of hemodynamic perturbations. Finally, the sedation protocol was not standardised across the study period, and variations in individual anaesthetist practice may have influenced both drug dosing and adverse event recognition and recording.

An important limitation of this study is the potential for confounding by indication in the analysis of adjunct sedative agents, particularly ketamine. Ketamine was administered at the discretion of the attending anaesthetist, most likely in patients perceived as more complex or at higher procedural or hemodynamic risk. Therefore, the observed association between ketamine use and adverse events may not reflect a direct causal effect, but rather the underlying risk profile of the patients in whom it was used. This association should be interpreted with caution. Furthermore, sedation was delivered by anaesthetists in training with a minimum of three years of experience under direct senior supervision, a staffing model that may not be representative of institutions relying on nurse-administered or endoscopist-directed sedation, or of centres without dedicated anaesthesia facilities for endoscopy; the findings should therefore not be extrapolated to such settings without caution. The absence of time-at-depth data, more specifically, cumulative duration at MOAA/S ≤ 2, precluded adjustment of propofol dose for depth of sedation achieved, and the relative contribution of dose versus depth to the observed adverse event rates cannot be fully disentangled from the available data.

## 5. Conclusions

Anaesthetist-delivered propofol-based sedation for ERCP is associated with a high incidence of predominantly low-severity adverse events when defined inclusively, but with a low rate of high-severity complications in a comorbid population. Propofol dose is the principal modifiable risk factor, demonstrating a consistent dose–response relationship across hemodynamic and respiratory outcomes. ASA physical status and age further identify patients at increased risk of clinically significant events requiring intervention. Ketamine use was associated with increased odds of adverse events; however, this association is likely confounded by indication and should not be interpreted as a direct causal effect. These findings support stepwise propofol titration guided by clinical sedation assessment, with avoidance of anticipatory dosing particularly in older patients and those with higher ASA scores, and highlight the safety of anaesthetist-led sedation in this setting.

## Figures and Tables

**Table 1:** Patient characteristics.

Variable	Value (Median [IQR] or *n* (%))
**Demographics**	
Age (years)	70.0 (58.8–77.0)
Sex, male	164 (42.3%)
**Clinical**	
ASA score	3.0 (2.0–3.0)
Pulmonary disease	61 (15.7%)
Hypertension	236 (60.8%)
**Procedural Characteristics**	
Time from admission to procedure (h)	48.0 (24.0–72.0)
Duration of procedure (min)	30.0 (20.0–40.0)
Wirsung cannulations	2.0 (2.0–2.0)
**Chronic Medication**	
ACE inhibitor use	154 (39.7%)
Statin use	73 (18.8%)
Anticoagulant use	42 (10.8%)
Antiplatelet use	26 (6.7%)
**Sedation**	
Propofol dose (mg)	150.0 (100.0–200.0)
Ketamine dose (mg)	0.0 (0.0–10.0)
Fentanyl dose (mg)	0.1 (0.1–0.1)
Midazolam dose (mg)	1.0 (0.0–2.0)

**Table 2:** Adverse physiological events: frequency, severity, and clinical interventions (*n* = 388).

Event	*n* (%)	Severity Metric, Median (IQR)	Intervention	Intervention Rate, *n* (%)	Intervention Dose, Median (IQR)
Arterial hypotension	94 (24.2%)	Min SBP 80 mmHg (76–88)	Ephedrine	88 (93.6%)	20 mg (10–30)
Arterial hypertension	79 (20.4%)	Max SBP 164 mmHg (156–171)	-	-	-
Tachycardia	55 (14.2%)	Max HR 108 bpm (103.5–110)	-	-	-
Desaturation	41 (10.6%)	Min SpO_2_ 88% (86–89)	Endotracheal intubation	3 (7.3%)	
Bradycardia	34 (8.8%)	Min HR 42bpm (40–44)	Atropine	34 (100%)	0.5 mg (0.50–0.88)

Events are ordered by descending frequency. Severity metric reflects the most extreme intraoperative value recorded for each patient with the event. Tachycardia and arterial hypertension had no paired pharmacological intervention variable in the dataset and are reported as physiological observations only. The absence of a recorded intervention for these events does not preclude clinical management via propofol dose reduction or procedure modification, which were not separately captured. SBP = systolic blood pressure; HR = heart rate; SpO_2_ = peripheral oxygen saturation; IQR = interquartile range.

**Table 3:** Univariate comparison of variables associated with anaesthetic adverse events.

Variable	No Event, *n* = 168	Anaesthetic AE, *n* = 220	*p*-Value
Age (years)	70.0 (56.8–76.2)	70.0 (61.8–77.0)	0.420
ASA score	2.0 (2.0–3.0)	3.0 (2.0–3.0)	**0.041**
Duration of procedure (min)	30.0 (20.0–35.5)	30.0 (25.0–40.0)	**<0.001**
Propofol dose (mg)	120.0 (100.0–170.0)	170.0 (110.0–242.5)	**<0.001**
Fentanyl dose (mg)	0.1 (0.1–0.1)	0.1 (0.1–0.1)	**0.001**
Midazolam dose (mg)	1.0 (0.0–2.0)	1.0 (0.0–2.0)	0.721
Ketamine dose (mg)	0.0 (0.0–0.0)	0.0 (0.0–10.0)	**0.012**
MOAA/S minimum	2.0 (2.0–2.0)	2.0 (2.0–2.0)	**0.017**
Wirsung cannulations (*n*)	2.0 (2.0–2.0)	2.0 (2.0–2.0)	0.967
Baseline HR (bpm)	70.0 (62.0–80.0)	72.5 (64.0–82.0)	**0.041**
Minimum HR (bpm)	63.5 (56.0–72.0)	64.0 (56.0–75.0)	0.739
Maximum HR (bpm)	78.0 (70.0–87.0)	81.0 (76.0–98.0)	<0.001
Baseline SBP (mmHg)	128.0 (115.0–140.0)	137.0 (120.0–151.5)	<0.001
Minimum SBP (mmHg)	108.0 (100.0–118.0)	105.0 (82.0–126.0)	0.056
Maximum SBP (mmHg)	130.5 (120.0–141.2)	138.0 (120.0–158.0)	<0.001
Time from admission to procedure (h)	48.0 (24.0–72.0)	48.0 (24.0–72.0)	0.579
Male sex	68 (40.5%)	96 (43.6%)	0.603
Pulmonary disease	21 (12.5%)	40 (18.2%)	0.167
Hypertension	96 (57.1%)	140 (63.6%)	0.233
ACE inhibitor use	61 (36.3%)	93 (42.3%)	0.278
Anticoagulant use	21 (12.5%)	21 (9.5%)	0.445
Antiplatelet use	9 (5.4%)	17 (7.7%)	0.471
Statin use	34 (20.2%)	39 (17.7%)	0.620
Ketamine used	34 (20.2%)	67 (30.5%)	**0.031**
Midazolam used	106 (63.1%)	139 (63.2%)	1.000
Malignant diagnosis	113 (67.3%)	145 (65.9%)	0.864
Angiocholitis	80 (47.6%)	93 (42.5%)	0.364
Stenting performed	22 (13.1%)	46 (20.9%)	**0.061**
Wirsung stent	3 (1.8%)	2 (0.9%)	0.656

Bold *p*-values indicate statistical significance (*p* < 0.05). Stenting performed value is borderline and was included into the regression. Mann–Whitney U test was used for continuous variables; Fisher’s exact test and chi-square test were used for categorical variables, as appropriate.

**Table 4:** Multivariate logistic regression analysis of predictors of anaesthetic adverse events. *n* = 387, Events = 108, Nagelkerke R^2^ = 0.43, McFadden R^2^ = 0.30, AIC = 341.3.

Variable	OR (95% CI)	*p*-Value
Age (years)	1.04 (1.01–1.07)	0.007
Male sex	1.14 (0.65–2.02)	0.650
ASA physical status	1.63 (1.07–2.49)	0.024
Propofol dose (per 10 mg)	1.20 (1.14–1.25)	<0.001
Ketamine use	2.18 (1.14–4.14)	0.018
Midazolam dose (mg)	1.03 (0.72–1.47)	0.882
Procedure duration (min)	0.99 (0.97–1.01)	0.254
Pulmonary disease	0.96 (0.46–2.01)	0.912
Hypertension (comorbidity)	1.22 (0.66–2.25)	0.531
Malignant diagnosis	1.11 (0.60–2.03)	0.740

Fentanyl was excluded from the model due to complete separation precluding stable coefficient estimation. *p*-values < 0.05 indicate statistical significance. Propofol dose entered as per-10 mg increment.

**Table 5:** Multivariate logistic regression analysis of individual anaesthetic adverse events.

Variable	OR	95% CI	*p*-Value
**Bradycardia (*n* = 388, events = 34, Nagelkerke R^2^ = 0.197)**			
Propofol (per 10 mg)	1.082	1.046–1.119	**<0.001**
Age (years)	1.027	0.995–1.061	0.104
ASA score	0.811	0.458–1.436	0.472
ACE inhibitor use	0.835	0.346–2.012	0.687
Hypertension	1.57	0.568–4.339	0.384
Ketamine use	0.851	0.331–2.19	0.738
Sex, male	0.966	0.445–2.096	0.930
**Hypotension (*n* = 387, events = 94, Nagelkerke R^2^ = 0.375)**			
Propofol (per 10 mg)	1.161	1.114–1.21	**<0.001**
Age (years)	1.02	0.996–1.045	0.109
ASA score	1.766	1.16–2.688	**0.008**
Procedure duration (min)	0.994	0.974–1.015	0.565
ACE inhibitor use	1.013	0.506–2.031	0.970
Hypertension	0.999	0.476–2.093	0.997
Ketamine use	1.847	0.975–3.498	0.060
Midazolam use	0.73	0.374–1.428	0.358
Pulmonary disease	1.669	0.817–3.408	0.160
Sex: male	1.042	0.587–1.852	0.887
**Desaturation (*n* = 387, events = 41, Nagelkerke R^2^ = 0.271)**			
Propofol (per 10 mg)	1.095	1.054–1.138	**<0.001**
Age (years)	1.015	0.985–1.046	0.328
ASA score	1.704	1.001–2.898	**0.049**
Procedure duration (min)	1.002	0.977–1.027	0.893
Pulmonary disease	2.469	1.059–5.755	**0.036**
Ketamine use	2.364	1.097–5.095	**0.028**
Midazolam use	1.068	0.440–2.593	0.885
Sex, male	1.476	0.704–3.092	0.302

Bold *p*-values indicate statistical significance (*p* < 0.05). Propofol dose entered as per-10 mg increment. Nagelkerke R^2^ reported per panel as a measure of model explanatory power. Models built using pre-specified variable selection; maximum one predictor per 10 events.

**Table 6:** High-severity outcomes stratified by anaesthetic adverse events.

Outcome	*n* Events	Rate (%)	with Anaesthetic AE (*n* = 220)	Without Anaesthetic AE (*n* = 168)	*p*-Value (Fisher’s Exact Test)
Flumazenil (reversal)	5	1.29	4 (1.8%)	1 (0.6%)	0.394
Procedure interrupted	2	0.52	2 (0.9%)	0 (0.0%)	0.508
Advanced airway (endotracheal intubation)	3	0.77	3 (1.4%)	0 (0.0%)	0.262
ICU admission	4	1.03	4 (1.8%)	0 (0.0%)	0.136
Death within 24 h	2	0.52	2 (0.9%)	0 (0.0%)	0.508
Overall death	2	0.52	2 (0.9%)	0 (0.0%)	0.508

## Data Availability

The data are encapsulated within this article. Further details can be obtained upon request from either the primary author or the corresponding author.
